# Avidity of influenza-specific memory CD8^+^ T-cell populations decays over time compromising antiviral immunity

**DOI:** 10.1002/eji.201242575

**Published:** 2012-10-16

**Authors:** Ian R Humphreys, Mathew Clement, Morgan Marsden, Kristin Ladell, James E McLaren, Kathryn Smart, James P Hindley, Hayley M Bridgeman, Hugo A van den Berg, David A Price, Ann Ager, Linda Wooldridge, Andrew Godkin, Awen M Gallimore

**Affiliations:** 1 Institute of Infection and Immunity, School of Medicine, Cardiff UniversityCardiff, UK; 2 Mathematics Institute, University of WarwickCoventry, UK

**Keywords:** CD8^+^ T cell, Cytotoxicity, IFN-γ, Influenza, Vaccination

## Abstract

Decline of cell-mediated immunity is often attributed to decaying T-cell numbers and their distribution in peripheral organs. This study examined the hypothesis that qualitative as well as quantitative changes contribute to the declining efficacy of CD8^+^ T-cell memory. Using a model of influenza virus infection, where loss of protective CD8^+^ T-cell immunity was observed 6 months postinfection, we found no decline in antigen-specific T-cell numbers or migration to the site of secondary infection. There was, however, a large reduction in antigen-specific CD8^+^ T-cell degranulation, cytokine secretion, and polyfunctionality. A profound loss of high-avidity T cells over time indicated that failure to confer protective immunity resulted from the inferior functional capacity of remaining low avidity cells. These data imply that high-avidity central memory T cells wane with declining antigen levels, leaving lower avidity T cells with reduced functional capabilities.

## Introduction

A goal of vaccination is to induce antigen-specific T cells that efficiently eliminate targets and generate memory T cells capable of conferring long-term protective immunity. The influenza surface antigens hemagglutinin and neuraminidase, which constitute the primary targets for antibody responses, vary substantially between virus strains due to antigenic drift. In contrast, the internal nucleo-protein (NP) antigen is highly conserved within and between influenza strains 1. Accordingly, influenza-specific T cells reactive to internal antigens such as NP afford cross-protection against multiple influenza subtypes and strains, suggesting that the induction of long-lived functional T cells with internal antigen specificity in response to infection or vaccination is critical for broad anti-influenza protection. Influenza-induced immunity against a serotype other than the primary infecting virus is termed heterosubtypic immunity 2,3. In mice, protective heterosubtypic immunity is predominantly mediated by CD8^+^ T cells 3,4. However, such protection afforded by pathogenic 5 or cold-adapted attenuated influenza 4 wanes over time, suggesting progressive impairment of the influenza-specific memory CD8^+^ T-cell response.

Loss of virus-specific T cells from the airways may be a critical factor underlying the decline of T-cell immunity to respiratory infections 6. Nonetheless, memory CD8^+^ T cells from the circulation and secondary lymphoid tissue (predominantly central memory cells) contribute to the maintenance of pulmonary T-cell immunity and are rapidly recruited to the site of virus re-challenge 6. Furthermore, although virus-specific CD4^+^ T-cell memory in secondary lymphoid tissue declines over time, memory CD8^+^ T-cell numbers remain stable 7. However, the capacity of these memory T cells to maintain the ability to respond to antigenic challenge and provide protection against re-infection is poorly understood.

Herein, we investigated whether the antiviral function of influenza-specific CD8^+^ T-cell memory is maintained over time. To study the capacity of these cells to confer long-term immunity in isolation, we utilized a mouse model of influenza infection in which responses to the immunodominant major histocompatibility complex (MHC) class I-restricted NP epitope were measured 8, and protective immunity was assessed after systemic challenge with recombinant vaccinia virus expressing the NP-derived peptide (rVV-NP). This model facilitates examination of a single influenza-specific memory CD8^+^ T-cell population quantitatively and qualitatively over time. By studying protective immunity to systemic virus challenge, we were able to assess the functional capacity of influenza-specific central memory CD8^+^ T-cell recall responses independently of the recruitment to and/or retention of memory cells in the airways.

## Results

### The capacity of memory CD8^+^ T cells for secondary expansion is maintained over time

**Figure 1:**
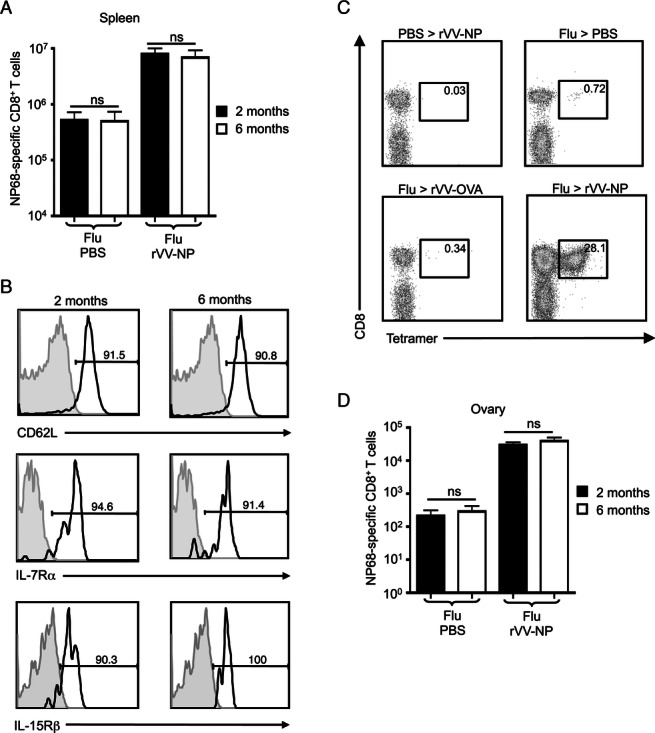
NP-specific CD8^+^ T cells maintain the capacity to expand following challenge. (A) C57BL/6 mice were infected with influenza and challenged with PBS or rVV-NP either 2 (closed bars) or 6 (open bars) months later. Five days later, tetramer-binding NP-specific CD8^+^ T cells in the spleen were quantified using the following calculation: total viable cells x%CD3^+^ x%tetramer^+^ cells. Data are shown as mean +SEM of 15–16 mice from three independent experiments. (B) Expression of CD62L (top), IL-7Rα (middle) and IL-15Rβ (bottom) by NP-specific tetramer-binding CD3^+^CD8^+^ cells was examined either 2 (left) or 6 (right) months after influenza infection. Shaded line, fluorescence minus one (FMO); solid line, antibody staining. Representative plots from two experiments comprising four to six mice per group per experiment are shown. (C) Mice were either mock-infected or infected with influenza. After 2 months, mock-infected mice were challenged with rVV-NP (top left), and influenza-infected mice were challenged with PBS (top right), rVV-OVA (bottom left) or rVV-NP (bottom right). Five days later, splenic NP-specific CD8^+^ T cells were detected by tetramer staining. Plots are gated on live CD3^+^ cells and%tetramer-binding CD3^+^CD8^+^ cells are shown. Data are the representative of three to six mice per group from more than six experiments. (D) Mice were treated as described in (A), and NP-specific CD8^+^ T cells in the ovaries were quantified as described in (A) prior to, and 5 days after, rVV-NP challenge. Data are shown as mean +SEMfor three to four mice and are the representative of three independent experiments. (A and D) ns = not significant (*p* > 0.05), as assessed by Student's *t*-test.

To examine the maintenance of protective influenza-specific CD8^+^ T cells, mice were infected with influenza and responses were characterized 2 or 6 months later. Numbers of splenic NP-specific peptide-MHC class I (pMHCI) tetramer-binding CD8^+^ T cells remained stable over time (Fig. 1A) with high levels of CD62L expression irrespective of the time point measured (Fig. 1B), indicating that persisting influenza-specific CD8^+^ T cells were predominantly central memory cells. IL-7 and IL-15 are critical for memory CD8^+^ T-cell homeostasis 9–11, and expression of IL-7R and IL-15R by NP-specific CD8^+^ T cells was comparable at 2 and 6 months after influenza infection (Fig. 1B).

To examine the expansion of these NP-specific CD8^+^ T cells, mice infected with influenza 2 or 6 months previously were challenged with rVV-NP. Following rVV-NP challenge, influenza-primed mice mounted an NP-specific recall CD8^+^ T-cell response in the spleen (Fig. 1C, bottom right) that was not induced by rVV expressing irrelevant protein (Fig. 1C, bottom left). Moreover, no expansion of NP-specific CD8^+^ T cells from the naive pool was evident at the time point (5 days postchallenge) measured (Fig. 1C, top left). Accordingly, this time point was selected to investigate recall responses because the vast majority of measureable NP-specific CD8^+^ T cells derive from the memory pool. Importantly, comparable expansion of NP-specific CD8^+^ T cells occurred irrespective of whether mice were challenged 2 or 6 months after influenza infection (Fig. 1A). Challenge of influenza-primed mice with rVV-NP also elicits NP-specific recruitment of CD8^+^ T cells into the ovary, which is a major site of rVV replication 8. Comparable numbers of NP-specific CD8^+^ T cells were detected in the ovaries irrespective of time after influenza infection (Fig. 1D).

### Protection afforded by NP-specific memory CD8^+^ T cells wanes over time

**Figure 2:**
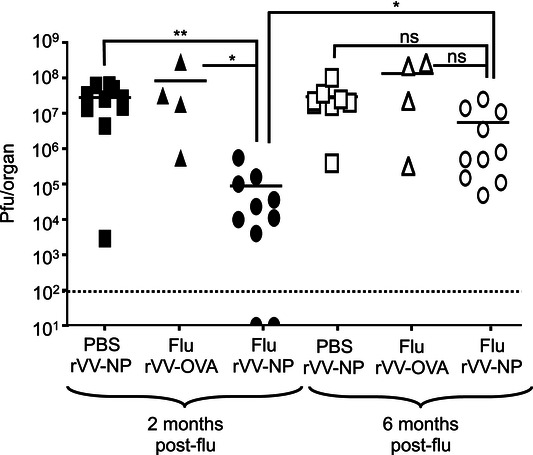
CD8^+^ T-cell-mediated protection from virus challenge decays over time. Mock-infected or influenza-infected mice were challenged with rVV-NP or rVV-OVA either 2 (closed symbols) or 6 (open symbols) months postinfection. Viral load in the ovaries was measured 5 days after rVV challenge. Individual mice from two experiments are shown; data are representative of 2/3 independent experiments. *p < 0.05, **p < 0.01, as assessed by the Mann–Whitney *U* test. ns = not significant.

As reported previously 8, rVV-NP (but not rVV-OVA) replication was inhibited in the ovaries when mice were challenged 2 months after influenza infection (Fig. 2). Thus, influenza-induced protection from rVV-NP challenge is NP specific. Strikingly, this protective effect was abrogated when rVV-NP challenge was delayed until 6 months after influenza infection (Fig. 2). These data demonstrate that systemic protective influenza-specific CD8^+^ T-cell memory wanes over time despite the maintenance of memory CD8^+^ T-cell numbers capable of secondary expansion and recruitment to the site of viral replication.

### Qualitative loss of functional virus-specific recall responses over time

**Figure 3:**
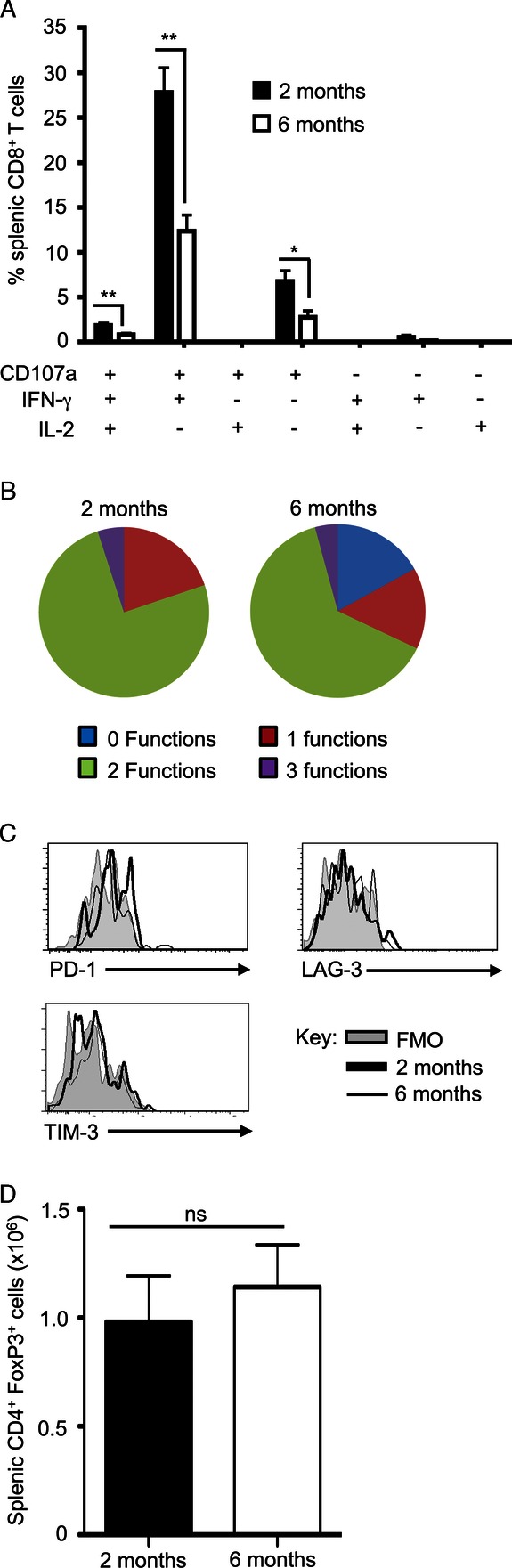
Delayed challenge of influenza-infected mice leads to impaired functionality of memory CD8^+^ T cells. (A and B) Influenza-infected mice were challenged with rVV-NP either 2 or 6 months postinfluenza infection. Five days later, CD107a mobilization and the production of IFN-γ and IL-2 by splenic CD8^+^ T cells was measured ex vivo in response to incubation with NP peptide (2 × 10^−6^M). (A) The percentage of peptide-specific CD107a^+/−^IFN-γ^+/−^IL-2^+/−^ cells within the total splenic CD8^+^ T-cell population was calculated by subtracting % positive in control-stimulated samples from % positive in peptide-stimulated samples. Results are expressed as mean + SEM of eight to ten mice per group and are representative of two separate experiments. *p < 0.05, **p < 0.01, as assessed by Student's *t*-test. (B) Pie charts displaying mean values of 0 to four functions across all permutations for NP-specific CD8^+^ T cells. (C) Splenocytes were isolated either 2 or 6 months after influenza infection and NP-specific tetramer-binding CD8^+^ T cells were assessed for PD-1, LAG-3, and TIM-3 expression. (D) CD4^+^FoxP3^+^ T cells were enumerated in splenocytes isolated either 2 or 6 months after influenza infection. (C and D) Data are shown as mean + SEM of four mice per group is shown and are representative of two experiments. ns = not significant.

To elucidate the mechanism(s) underpinning the loss of protective CD8^+^ T-cell memory, we studied the functional profiles of T cells elicited by rVV-NP challenge 2 or 6 months after influenza infection. Two months after influenza infection, rVV-NP challenge induced the accumulation of functional CD8^+^ T cells capable of ex vivo degranulation and/or expression of IFN-γ and/or IL-2 (Fig. 3A and B). These NP-specific CD8^+^ T cells were primarily polyfunctional, with CD107a^+^IFN-γ^+^ cells representing the dominant population (Fig. 3A and B). Critically, despite inducing equivalent numbers of memory CD8^+^ T cells after 6 months (Fig. 1A and D), delayed rVV-NP challenge induced the accumulation of far fewer functional NP-specific CD8^+^ T cells (Fig. 3A and B). By comparing the percentage of NP-specific tetramer-binding cells and the percentage of cells exhibiting at least one peptide-specific response, approximately 20% of NP-specific CD8^+^ T cells elicited by rVV-NP 6 months after influenza priming displayed no detectable function. In contrast, all NP-specific memory CD8^+^ T cells elicited by rVV-NP 2 months after influenza infection exhibited one or more functions. Although CD107a^−^IFN-γ^−^IL-2^−^ cells may express other proinflammatory cytokines such as TNF and MIP-1β 12, these data demonstrate that a significant proportion of the recall response elicited by delayed rVV-NP challenge does not exhibit ex vivo degranulation or the production of cytokines important for antiviral memory T-cell responses (IL-2 and IFN-γ). Thus, the ability of systemic CD8^+^ T-cell memory to elicit a functional antiviral recall response wanes over time following initial T-cell priming.

### Reduced T-cell receptor avidity of memory CD8^+^ T cells elicited following delayed challenge

Next, we investigated the potential mechanism(s) underpinning the progressive loss of functional memory CD8^+^ T-cell responses. No upregulation of PD-1, LAG-3, or TIM-3 was apparent on NP-specific memory CD8^+^ T cells either 2 or 6 months after influenza infection (Fig. 3C), and numbers of FoxP3^+^ regulatory T (Treg) cells were comparable (Fig. 3D). These data argue against a role for co-inhibitory molecules and Treg cells as modulators of the CD8^+^ T-cell recall response in this model.

**Figure 4:**
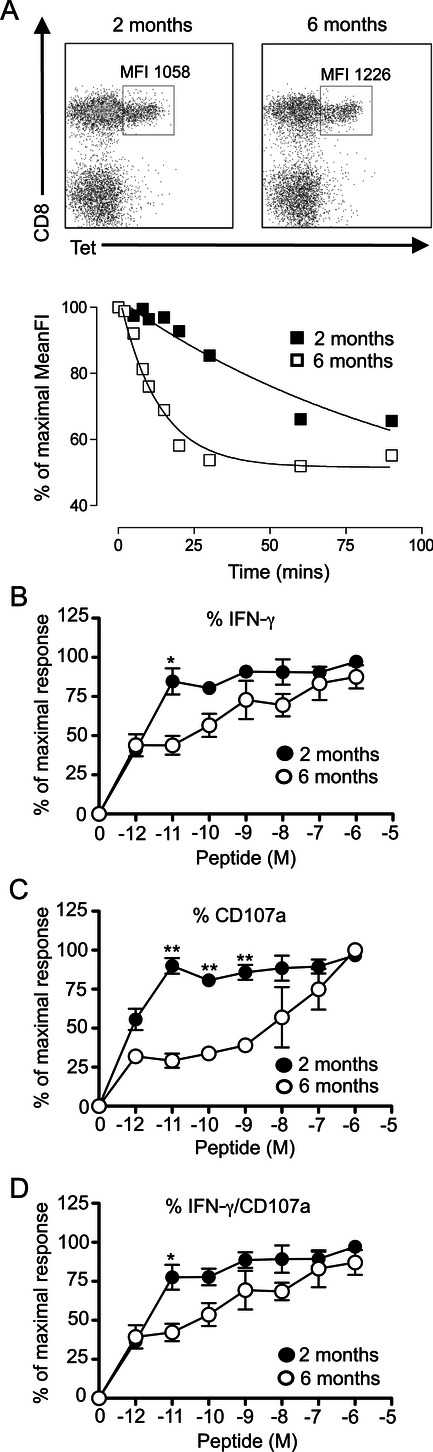
Reduced memory CD8^+^ T-cell avidity following delayed rVV-NP challenge. (A) Representative bivariate flow cytometry plots showing tetramer staining of NP-specific CD8^+^ T cells induced by rVV-NP either 2 (top left) or 6 (top right) months postinfluenza infection. Tetramer decay from the surface of NP-specific CD8^+^ T cells is shown below. Results are expressed as % maximal binding and represent two independent experiments. In the assay shown, half-life values for tetramer binding were 71.35 min (2 months) and 8.95 min (6 months). (B-D) Influenza-infected mice were challenged with rVV-NP either 2 or 6 months postinfluenza infection. Splenocytes were isolated 5 days later and total (B) IFN-γ^+^, (C) CD107a^+^ or (D) CD107a^+^IFN-γ^+^ NP-specific CD8^+^ T-cell responses were measured across a range of cognate peptide concentrations. Results are expressed as % maximal response. (B-D) Data are shown as mean ± SEM of three mice per group and are representative of two independent experiments. *p < 0.05, **p < 0.01, as assessed by Student's *t*-test.

The avidity of surface-expressed TCRs for MHCI-peptide complexes is associated with CD8^+^ T-cell antiviral function 13–18 and influences cross-reactive protection afforded by influenza-specific CD8^+^ T cells 19. Although saturating concentrations of the H-2D^b^/NP68 tetramer were bound comparably by memory CD8^+^ T cells isolated 5 days after rVV-NP challenge irrespective of time after influenza priming (Fig. 4A), tetramer decay experiments demonstrated a striking reduction in TCR off-rate for NP-specific CD8^+^ T cells expanded by rVV-NP challenge 6 months postinfluenza infection compared with the corresponding cells expanded 2 months postinfluenza infection (Fig. 4A). Indeed, in two repeat experiments, the half-life of tetramer binding 6 months postinfection was reduced by 7.6–8-fold compared with the 2-month time point (Fig. 4A and data not shown). These results suggest that the loss of antiviral functionality (defined by CD107a, IFN-γ, and IL-2; Fig. 3A and B) by some NP-specific CD8^+^ T cells following delayed challenge likely represents reduced TCR avidity for antigenic H-2D^b^/NP68 complexes. Furthermore, CD107a^+^ and/or IFN-γ^+^ NP-specific CD8^+^ T cells derived after delayed challenge exhibited an impaired ability to respond to low concentrations of cognate peptide in vitro compared with the corresponding cells isolated 2 months after rVV-NP challenge (Fig. 4B-D), with a 2–4 log reduction in functional sensitivity (Table 1). Collectively, these data demonstrate that the antigen sensitivity of influenza-specific memory CD8^+^ T-cell populations wanes over time and impairs protective immunity to secondary challenge.

**Table:** Reduced functional avidity of memory CD8^+^ T cells after delayed rVV-NP challenge

	Months postinfluenza infection	*p*EC_50_^a^
IFN-γ^+^	2	11.51
	6	9.54
CD107a^+^	2	11.97
	6	7.78
IFN-γ^+^/CD107a^+^	2	11.24
	6	9.26

a) *p*EC_50_ of data displayed in Figure 4B–D were estimated as described in the *Materials and methods*.

## Discussion

Herein, we demonstrate that functional decay within the systemic virus-specific CD8^+^ T-cell recall response over time is due to the loss of high avidity memory CD8^+^ T cells. A number of factors may govern protective memory to respiratory viral infections, in particular the retention of memory T cells within the airways and recruitment of peripheral T cells to the pulmonary site of challenge 6. Our data imply that maintenance of the functional capacity conferred by high avidity cells within the memory T-cell pool may also contribute. Although our study focused on the analysis of memory T-cell responses to a single influenza peptide determinant and measured recall after challenge with a recombinant vaccinia virus, these findings are most likely relevant to rechallenge with influenza virus strains that share conserved internal antigens 5.

Notably, we also report a correlation between the loss of CD8^+^ T cell mediated protective immunity and impaired ex vivo T-cell function. The functional decay of influenza-specific memory CD8^+^ T cells was predominantly reflected by diminished degranulation and/or IFN-γ production. Polyfunctionality is thought to be an important correlate of protective antiviral T-cell activity. For example, polyfunctional CD8^+^ T cells are more readily measured in HIV nonprogressors and effectively suppress HIV replication in vitro 12,13. In our system, we observed significant reductions in functional T-cell numbers across all effector profiles, thereby demonstrating that polyfunctional T cells were not exclusively lost over time. It is possible, however, that the loss of polyfunctional cells contributed most to the loss of protective immunity. Indeed, the majority of NP-specific CD8^+^ T cells performed at least two functions (degranulation and IFN-γ production) at 2 and 6 months postinfection. In a previous study, HIV-specific CD8^+^ T cells with higher levels of TCR avidity and antigen sensitivity also exhibited greater polyfunctionality 12. Conversely, TCR avidity and polyfunctionality were not directly correlated in a study of influenza-specific CD8^+^ T cells 20. Although we observed a significant overall decline in NP-specific CD8^+^ T-cell avidity over time, it remains unclear whether this relates to a specific loss of polyfunctional CD8^+^ T cells.

It is uncertain whether CD8^+^ T-cell avidity correlates with antiviral potential under all conditions. Indeed, a recent in vitro study suggested that lower TCR avidity may increase the serial killing efficacy of influenza-specific CD8^+^ T cells by reducing target cell elution time 21. Such a finding may be relevant when all T cells within a defined population exceed a certain avidity threshold. However, the results presented herein and those reported previously using the Lymphocytic choriomeningitis model are consistent with the hypothesis that, certainly in the case of long-lived CD8^+^ T-cell memory, high avidity interactions confer the most antiviral protection 15, perhaps through enhanced polarization of cytotoxic machinery to the immunological synapse 21. Similarly, high-avidity T cells are thought to confer superior protection in other systems 12,18,22.

Although our experiments focused on T-cell populations elicited during recall responses, the data imply that high avidity memory CD8^+^ T cells are lost over time prior to rechallenge. It is not clear why this occurs. A number of factors influence avidity, including MHC-CD8 interactions 23 and the recruitment of signaling molecules to the TCR-CD3 complex 24, and the regulation of these processes in memory CD8^+^ T cells over time is not understood. Importantly, TCR sequence and subsequent affinity for peptide-MHC is an important determinant of avidity for some, but not all, influenza-specific CD8^+^ T-cell populations 25. Interestingly, independent studies examining influenza antigens in infected mice have revealed that virus persists in lung-draining lymph nodes for up to 70 days following infection 26,27,28. Antigen persistence promotes the accumulation of IFN-γ^+^ influenza-specific T cells in lung-draining lymph nodes 26. Declining antigen levels (based on published data 26,27,28) correspond temporally with the loss of functional influenza-specific CD8^+^ T cells in our model. It is therefore possible that cessation of antigen presentation influences long-term systemic functional antiviral CD8^+^ T-cell immunity. Indeed, it has been reported that selection for high avidity CD8^+^ T cells occurs in the case of viruses that establish persistent infections 29,30. Thus, the waning of high avidity CD8^+^ T cells in our model may be due to the loss of influenza antigen presentation 2–3 months following influenza infection.

Based on the above considerations, it is tempting to speculate that high avidity clonotypes may be “diluted” during homeostatic memory T-cell turnover in the absence of antigen 31 or as a result of nonmalignant T-cell expansions 32. Indeed, when we analyzed the clonotypic composition of NP-specific CD8^+^ T-cell populations 5 days after challenge with rVV-NP in mice infected with influenza 2 or 6 months previously, we observed that the TCR repertoire of each individual mouse was largely distinct, although some subdominant public TCRs were observed within time points (Supporting Information Table 1). Unfortunately, a longitudinal analysis within individual mice was not possible in this model. Nevertheless, these data support the hypothesis that the NP-specific CD8^+^ TCR repertoire alters over time, which could reflect the loss of high avidity cells. Interestingly, in a model of respiratory Sendai virus infection, the memory CD8^+^ T-cell pool acquired an enhanced proliferative capacity over time that was associated with the enrichment of highly proliferative CD62L^hi^ cells 33. In further studies, it would be interesting to determine whether low and high avidity NP-specific CD8^+^ T cells differ in their capacity to proliferate and whether this could contribute to a progressive shift towards low avidity T cells in the absence of ongoing antigenic drive. Irrespective of the exact mechanism by which avidity decays within the memory CD8^+^ T-cell population, our data imply that persistent antigen may be critical for the induction and maintenance of long-lived protective antiviral T-cell responses, in line with previous reports 34,35.

In summary, the data presented herein suggest that the loss of memory CD8^+^ T-cell function may contribute to waning heterosubtypic immunity against influenza. Furthermore, these results imply that the induction of effective long-lived T-cell memory, which requires that antiviral functional capacities are preserved over time, may depend on the presence of persistent antigen. Finally, this study demonstrates that effective T-cell memory should be assessed not only quantitatively, but also using qualitative parameters, including the ability to respond to challenge and exert antiviral function.

## Materials and methods

### Viral infection

Experiments were conducted according to UK Home Office guidelines. C57BL/6 mice were infected intranasally under inhalation anesthetic with 100 pfu influenza (H17) and, 2 or 6 months later, injected intraperitoneally with PBS or 2 × 10^6^ pfu rVV expressing OVA (rVV-OVA 36) or rVV-NP 37. Viral load in ovaries was enumerated 5 days later 8,38.

### Flow cytometry

Ovarian and splenic single-cell suspensions were incubated with LIVE/DEAD fixable violet (ViViD; Life Technologies), Fc block (eBioscience), and H-2D^b^/NP68 tetramer (ASNENMDAM). Cells were subsequently stained with αCD3-PerCP-Cy5.5 and αCD8-allophycocyanin-H7 (BD Pharmingen). In some experiments, aCD62L-Pe-Cy7 (Abcam), αCD122-FITC, aCD127-Pacific Blue, αPD-1-FITC, αTIM-3-PE, and αLAG-3-PE (all eBioscience) were used. In other experiments, cells were stained with αCD4-Pacific Blue (BD Pharmingen) and αFoxP3-PE according to the manufacturer's instructions (eBioscience). For functional analyses, frozen splenocytes were thawed, rested overnight, and stimulated with peptide at the depicted concentrations; degranulation and cytokine production were assessed by flow cytometry as described previously 39. Data were acquired using a FACSCanto II flow cytometer (BD Biosciences) and analyzed with FlowJo version 8.3 (TreeStar Inc.).

### pMHCI tetramer decay assay

Tetramer decay experiments were performed as described previously 29. Briefly, 1 × 10^6^ cells were stained with 100 μg/mL of PE-conjugated H-2D^b^/NP68 tetramer, washed and resuspended in two separate aliquots, then stained with ViViD, αCD45R/B220-PE-Cy7 (Biolegend), and αCD8-PerCP (BD Pharmingen). An excess of unlabeled H-2D^b^/NP68 tetramer was added to one aliquot of cells to block tetramer rebinding; cells were harvested at serial time points as depicted and analyzed by flow cytometry. The remaining sample was analyzed at the zero time point.

### Estimation of *p*EC_50_

Functional sensitivity was quantified as described previously 40. The *p*EC_50_ value is defined as the negative base-10 logarithm of the concentration at which 50% response efficacy is obtained. Accordingly, increases in functional sensitivity translate into increases in the pEC_50_ value.

### TCR clonotyping

Viable H-2D^b^/NP68 tetramer-binding CD3^+^CD8^+^ cells (5000 per population) were sorted at more than 98% purity using a customized FACSAria II flow cytometer (BD Biosciences) and clonotypic analysis was conducted using a template-switch anchored RT-PCR as described previously 41. The International ImMunoGeneTics information system (IMGT®) nomenclature was used to assign *TRB* gene usage 42.

## Abbreviations

MHCI: major histocompatibility complex class I
NP: nucleoprotein

## Notes

Disclaimer: Supplementary materials have been peer-reviewed but not copyedited.

Supporting Information Table 1. The clonotypic composition of NP-specific CD8^+^ T-cell populations is distinct between mice and time-points. (A and B) Influenza-primed mice were challenged with rVV-NP either 2 (A) or 6 (B) months after influenza infection. TRBV and TRBJ usage, CDR3β amino acid sequence and relative frequency are shown for individual NP-specific clonotypes within viable tetramer-binding CD3^+^CD8^+^ cell populations isolated by flow cytometric sorting 5 days after rVV-NP challenge. Two mice/time-point are shown.
