# Supplementary material for: Avidity of influenza-specific memory CD8+ T-cell populations decays over time compromising antiviral immunity
Source: Eur J Immunol. 2012 Oct 16;42(12):3235–42. doi: 10.1002/eji.201242575 (PMC3657127; doi:10.1002/eji.201242575)
Supplement: Supporting Information Table 1. — The clonotypic composition of NP-specific CD8+ T-cell populations is distinct between mice and time-points. (A and B) Influenza-primed mice were challenged with rVV-NP either 2 (A) or 6 (B) months after influenza infection. TRBV and TRBJ usage, CDR3β amino acid sequence and relative frequency are shown for individual NP-specific clonotypes within viable tetramer-binding CD3+CD8+ cell populations isolated by flow cytometric sorting 5 days after rVV-NP challenge. Two mice/time-point are shown. [file eji0042-3235-sd1.pdf]

European Journal of Immunology

Supporting Information

for

DOI 10.1002/eji.201242575

Ian R. Humphreys, Mathew Clement, Morgan Marsden, Kristin Ladell, James E. McLaren, Kathryn Smart, James P. Hindley, Hayley M. Bridgeman, Hugo A. van den Berg, David A. Price, Ann Ager, Linda Wooldridge, Andrew Godkin and Awen M. Gallimore

Avidity of influenza-specific memory CD8$^+$ T-cell populations decays over time compromising antiviral immunity

© 2012 WILEY-VCH Verlag GmbH & Co. KGaA, Weinheim

A

TRBV	CDR3	TRBJ	Freq (%)
31	CAWSLRVGQNTLY	2-4	30.88
3	CASSPRTGSQNTLY	2-4	17.65
20	CGARGDWGGNYAEQF	2-1	13.24
13-1	CASSARTANTEVF	1-1	10.29
13-3	CASSDNYNSPLY	1-6	10.29
19	CASSPRHSANTEVF	1-1	4.41
13-3	CASSDVGQNQAPL	1-5	4.41
31	CAWSLVGYEQY	2-7	2.94
20	CGARGDWGGNYAHQF	2-1	1.47
2	CASSSRQGNSGNTLY	1-3	1.47
17	CASSPRRGRGDTQY	2-5	1.47
13-3	CASSEVQNTEVF	1-1	1.47

TRBV	CDR3	TRBJ	Freq (%)
17	CASSRRIFYEQY	2-7	16.67
3	CASSSRTGGQNTLY	2-4	13.64
13-3	CASSETGWEQY	2-7	10.61
13-1	CASSERTEVF	1-1	10.61
16	CASSPLTGTSYEQY	2-7	6.06
13-1	CASSDPGQNYEQY	2-7	6.06
13-3	CASRLDRGYEQY	2-7	6.06
16	CASSSRRGHSGNTLY	1-3	4.55
2	CASSSRQGNSGNTLY	1-3	4.55
13-3	CASSSVQGVEQY	2-7	4.55
16	CASSSRQGASGNTLY	1-3	1.52
16	CASSTGGLGGRGTLY	2-3	3.03
16	CASSSRQGVSGNTLY	1-3	3.03
16	CASSSRTGASGNTLY	1-3	1.52
16	CASSSRRGHPGNTLY	1-3	1.52
13-3	CASSEWDRGGQNTLY	2-4	1.52
13-2	CASGDAGQDYNSPLY	1-6	1.52
19	CASSIGRGQGNTLY	1-3	1.52
16	CASSLRQGMSQAPL	1-5	1.52

B

TRBV	CDR3	TRBJ	Freq (%)
12-1	CASSPRTGGDEQY	2-7	25.45
5	CASSQAGYEQY	2-7	18.18
3	CASSSRTGGRGDTQY	2-5	14.55
16	CASSPRQGARAEQF	2-1	14.55
14	CASSFRLGGRGAEQF	2-1	10.91
19	CASSILGGGAETLY	2-3	3.64
3	CASSPRTGGANTQY	2-5	3.64
17	CASSRDRSDTQY	2-5	3.64
16	CASSPRQGARAGQF	2-1	1.82
3	CASSPPGQNQAPL	1-5	1.82
13-1	CASRRDKSYEQY	2-7	1.82

TRBV	CDR3	TRBJ	Freq (%)
12-1	CASSLRQGARGNTLY	1-3	51.32
19	CASSIMGGGAETLY	2-3	21.05
17	CASRKRTGQSYEQY	2-7	10.53
16	CASSSRQGRAGNTLY	1-3	9.21
17	CASSRRDRGQDTQY	2-5	2.63
13-3	CASRDWGSQNTLY	2-4	2.63
19	CASSILGGGAETLY	2-3	1.32
16	CASSPRQGARAEQF	2-1	1.32

Supporting Information **Table 1. The clonotypic composition of NP-specific CD8+ T-cell populations is distinct between mice and time-points**. (A and B) Influenza-primed mice were challenged with rVV-NP either 2 (A) or 6 (B) months after influenza infection. TRBV and TRBJ usage, CDR3β amino acid sequence and relative frequency are shown for individual NP-specific clonotypes within viable tetramer-binding CD3+CD8+ cell populations isolated by flow cytometric sorting 5 days after rVV-NP challenge. Two mice/time-point are shown.
